# Supplementary material for: CD4+ helper T cells endow cDC1 with cancer-impeding functions in the human tumor micro-environment
Source: Nat Commun. 2023 Jan 13;14:217. doi: 10.1038/s41467-022-35615-5 (PMC9839676; doi:10.1038/s41467-022-35615-5)
Supplement: Supplementary file 1 — Supplementary Figures and Legends [file 41467_2022_35615_MOESM1_ESM.pdf]

CD4+ helper T cells endow cDC1 with cancer-impeding functions in the human tumor micro-environment

Xin Lei[1,2], Indu Khatri[1,$], Tom de Wit[1,2,$], Iris de Rink[3], Marja Nieuwland[3], Ron Kerkhoven[3], Hans van Eenennaam[4], Chong Sun[5], Abhishek D. Garg[6], Jannie Borst[1,2,#,*] and Yanling Xiao[1,2,*]

[1]Department of Immunology and [2]Oncode Institute, Leiden University Medical Center, Leiden, The Netherlands; [3]Genomics Facility, The Netherlands Cancer Institute, Amsterdam, The Netherlands; [4]Aduro Biotech Europe B.V., Oss, The Netherlands; [5]Immune Regulation in Cancer, German Cancer Research Center, Heidelberg, Germany and [6]Laboratory of Cell Stress & Immunity, Department of Cellular & Molecular Medicine, KU Leuven, Leuven, Belgium

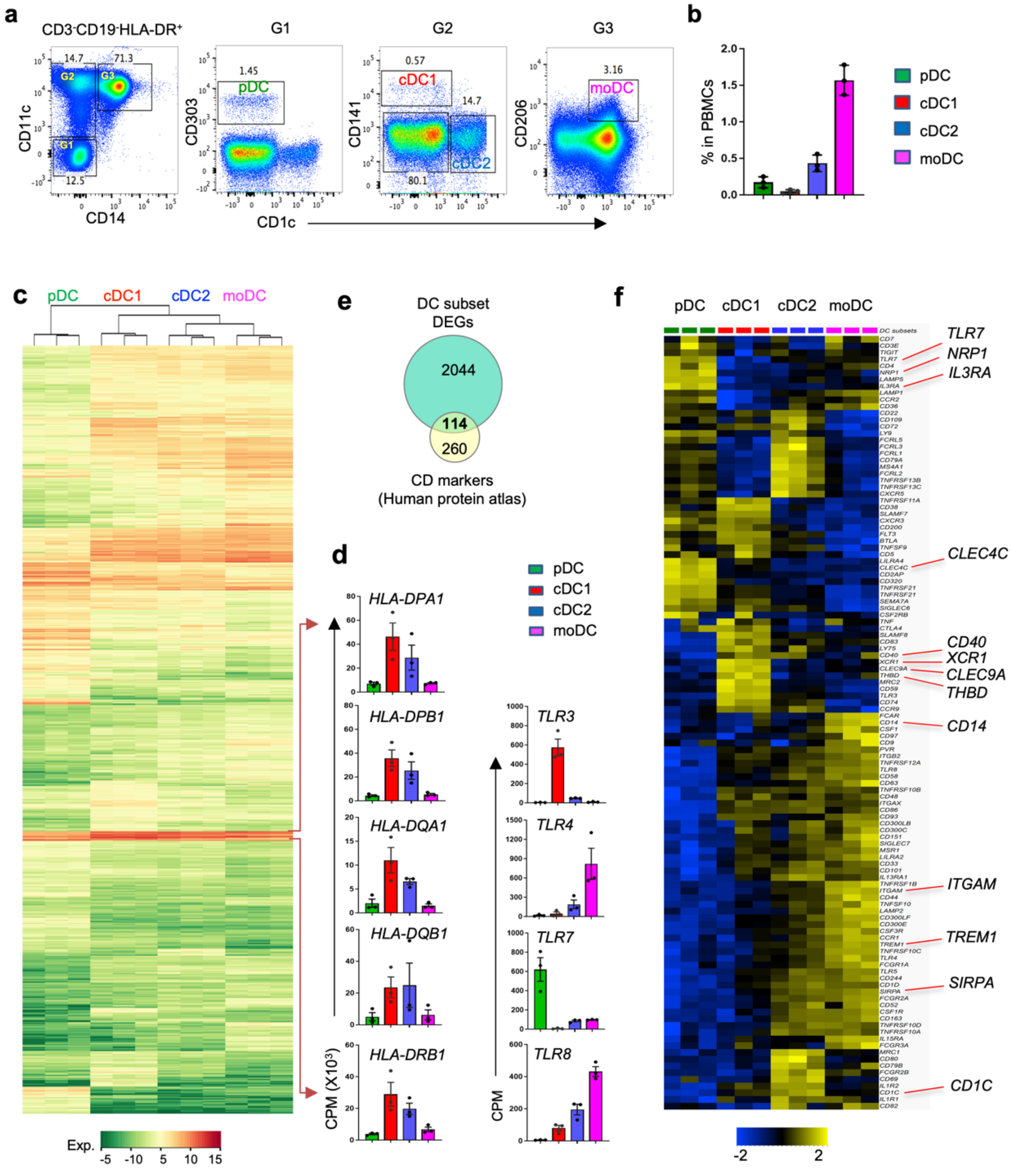

Supplementary Figure 1. Validation of human *ex vivo* pDC, cDC1, cDC2 and moDC subsets by transcriptomics. CD11c$^-$CD303$^+$ pDC, CD11c$^+$CD141$^+$ cDC1, CD11c$^+$CD1c$^+$ cDC2 and CD11c$^+$CD14$^+$ moDC were purified by flow cytometry from fresh PBMC of three healthy donors for bulk mRNA-sequencing. (**a**) Gating strategy for DC subset sorting. (**b**) Frequency (%) of each DC subset within total live PBMC (n=3). Data are derived from three independent biological replications (n=3) and are shown as means ± SEM. (**c-h**) Results of bulk mRNA-seq. Sequence reads were aligned to the Ensembl gene set *Homo sapiens* GRCh37.66. (**c**) Heatmap depicting 2158 differentially expressed genes (DEGs) between DC subsets (p-value <0.01; $\log_2$-fold change >2 or <-2). (**d**) Bar charts depicting mRNA expression of indicated genes in each DC subset based on read counts per million (CPM). Data are derived from three independent biological replications (n=3) and are shown as means ± SEM. (**e**) Venn diagram indicating that among the 2158 DEGs, 114 encoded CD markers as listed by Human Protein Atlas (out of a total of 374 CD markers). (**f**) Heatmap of the 114 CD markers that were differentially expressed between the DC subsets.

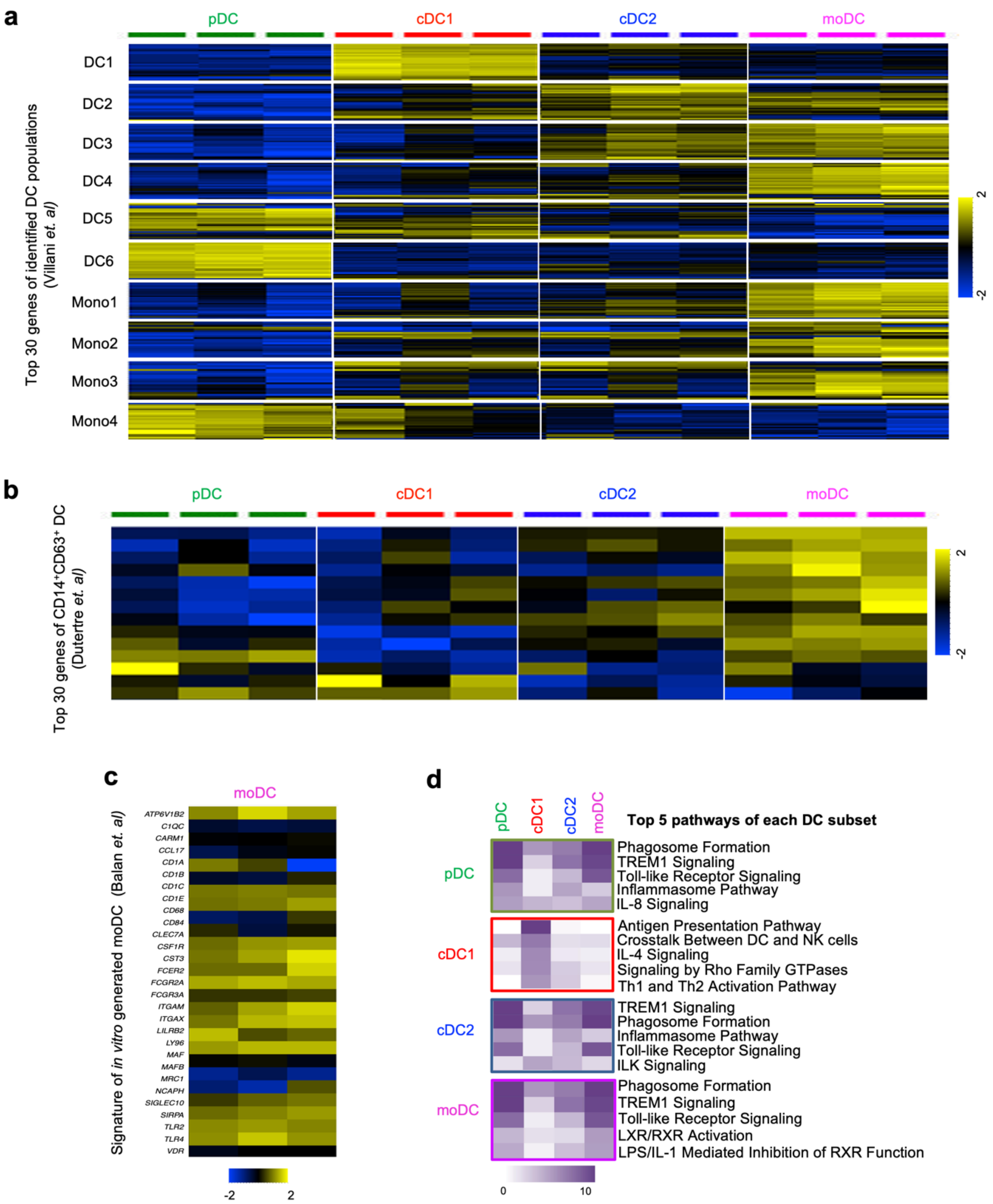

Supplementary Figure 2. Further validation of human *ex vivo* pDC, cDC1, cDC2 and moDC subsets. Analysis is based on transcriptome data of DC subsets obtained as outlined in Extended Data Figure 1. (**a**) Heatmap of top 30 signature genes of DC and monocyte subtypes/states from single cell transcriptome data of Villani *et al.*[20] as expressed by our DC subsets. (**b**) Heatmap of top 30 signature genes of $CD14^{+}CD63^{+}$ DC from single cell transcriptome data of Dutertre *et al.*[21] as expressed by our DC subsets. (**c**) Heatmap of indicated signature genes of *in vitro* generated moDC from bulk transcriptome data of Balan *et al.*[22] as expressed by our *ex vivo* moDC. (**d**) Top five upregulated pathways in each DC subset as derived from Ingenuity Pathway Analysis (IPA).

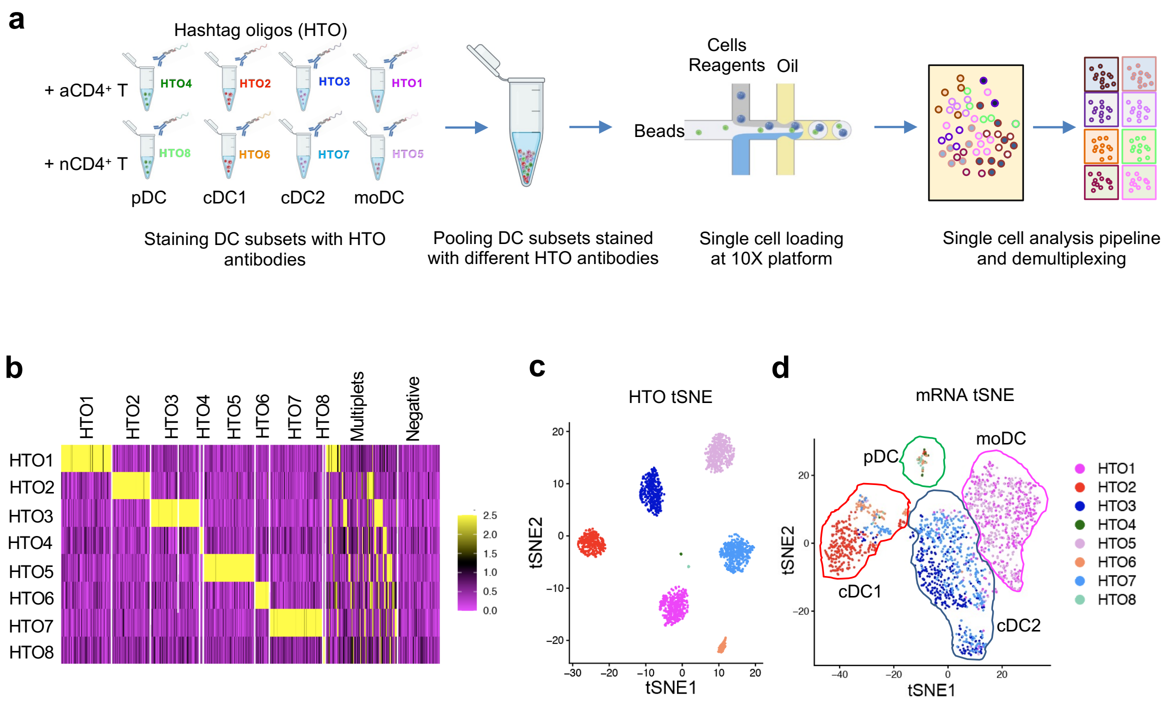

Supplementary Figure 3. Detecting the human DC response to CD4⁺ T-cell help by Hashtag scRNA-seq. *Ex vivo* pDC, cDC1, cDC2 and moDC were cultured with activated- or naive CD4⁺ T-cells and next labeled with HTO 1-8, pooled together in equal proportion, then loaded on a 10X Genomics platform for scRNA-seq. (**a**) Illustration of experimental design for scRNA-seq. (**b**) Heatmap depicting scaled (*z*-scores) normalized detection of HTO. Multiplets contain more than one different HTO. Negative populations contain no HTO. (**c**) tSNE plot of the singlets based on HTO detection depicts the separation between HTO 1-8. Cells were colored based on their specific HTO labeling. (**d**) tSNE plot of CD3⁻ DC single-cell expression profile based on top 2000 variable genes. DC subsets from conditions outlined in (**a**) are depicted in the relevant HTO color.

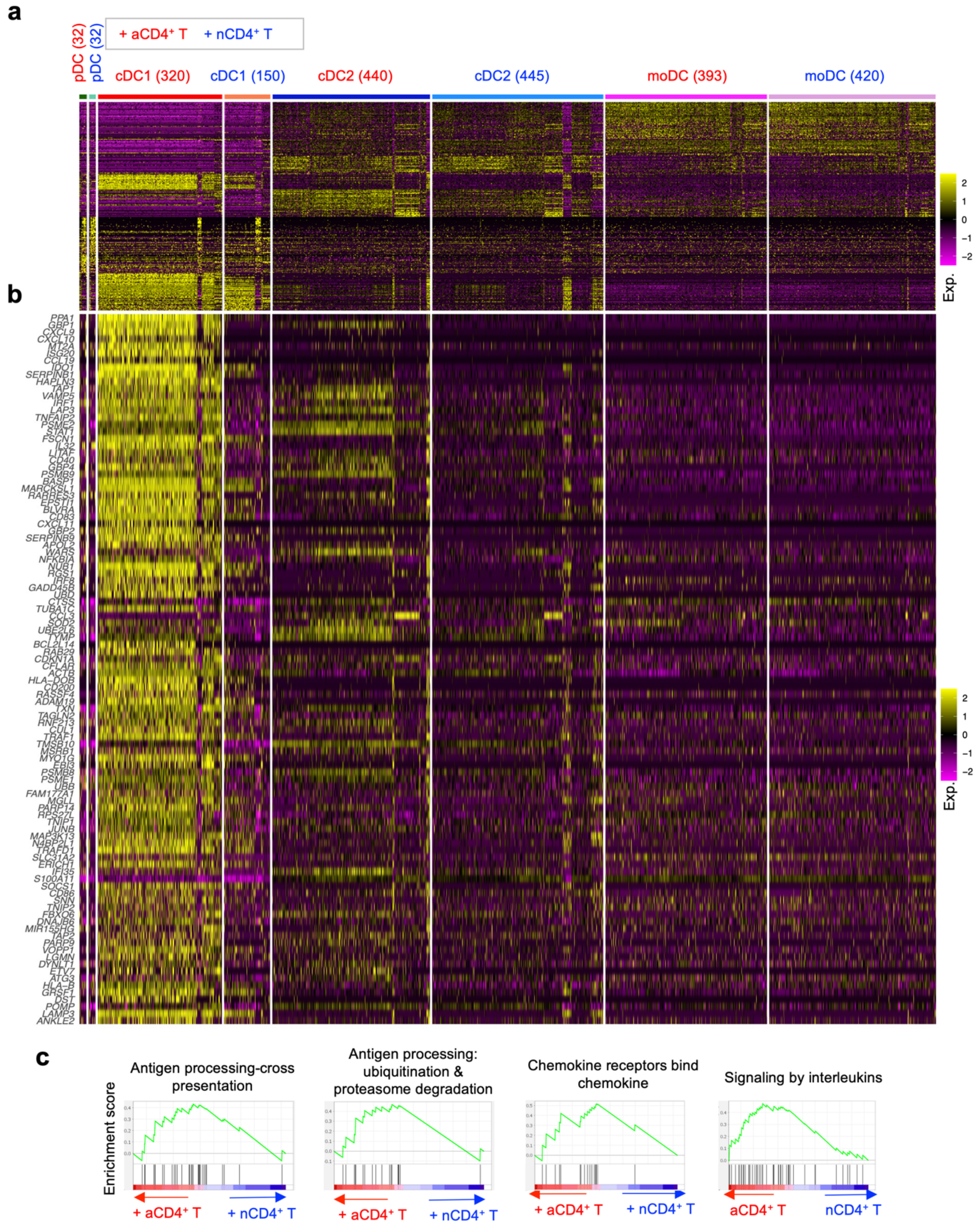

Supplementary Figure 4. Identification of human cDC1 help signature by Hashtag scRNA-seq. (**a**) Heatmap depicting scaled expression values of top 100 DEGs per HTO cluster. Cell number per cluster that passed quality control is indicated in brackets. (**b**) Heatmap depicting the expression of top 100 upregulated DEGs in the cDC1 help signature in each DC subset. (**c**) Gene set enrichment analysis (GSEA) of the 577 DEG in the cDC1 help signature denoting enrichment of indicated Reactome pathways.

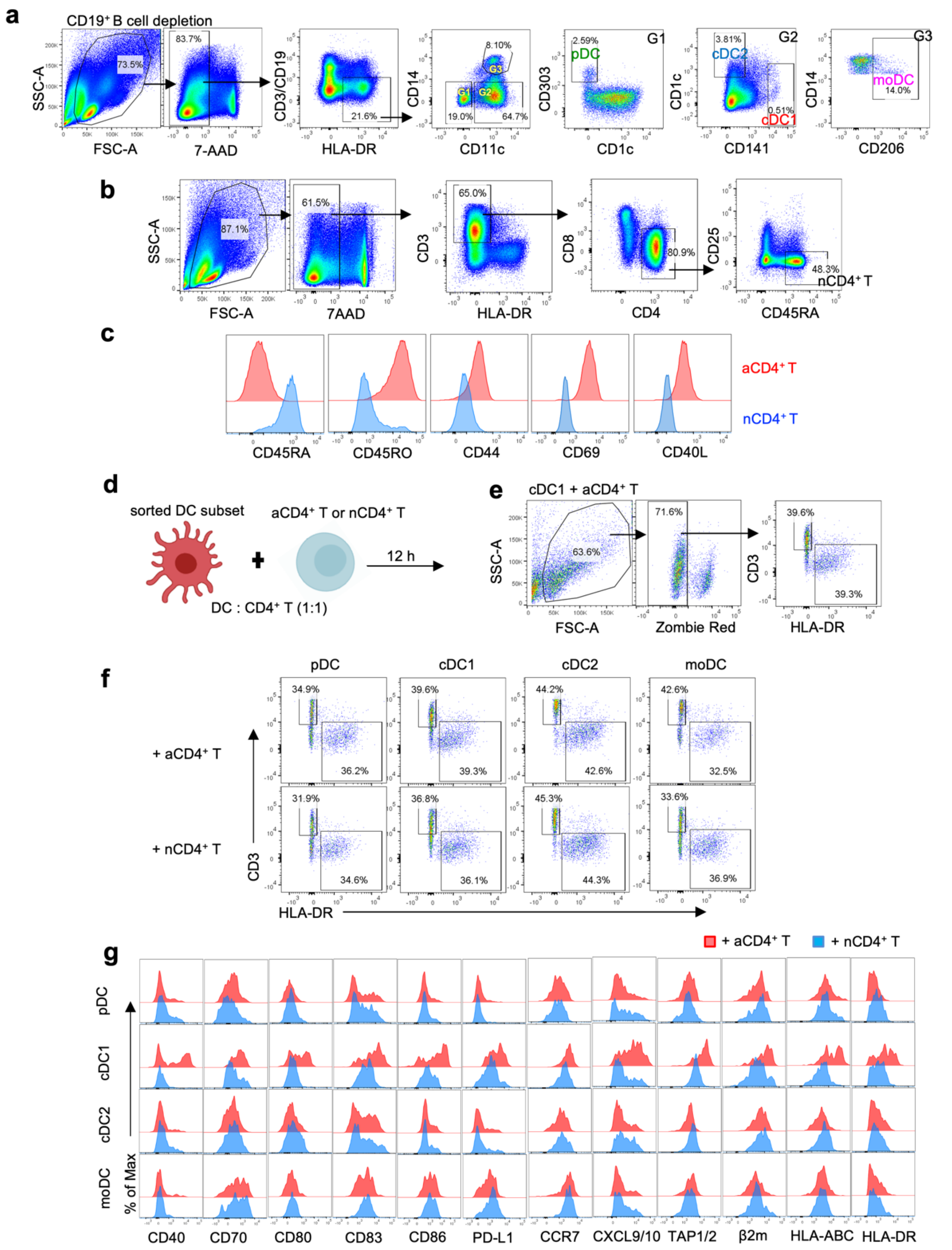

Supplementary Figure 5. Method for validation of the human cDC1 help signature. Key molecules of the cDC1 help signature were flow cytometrically validated after co-culturing *ex vivo* pDC, cDC1, cDC2 or moDC with activated- or naive CD4$^+$ T-cells. (**a, b**) Gating strategies for sorting (**a**) pDC, cDC1, cDC2 or moDC and (**b**) conventional naïve CD4$^+$ T-cells from cryopreserved PBMC. (**c**) Expression (MFI) of indicated markers on activated (a)- or naive (n) CD4$^+$ T-cells. (**d**) Illustration of the DC-CD4$^+$ T-cell co-culture. (**e**) Gating strategy for flow cytometric analysis of DC after co-culture with CD4$^+$ T-cells. (**f**) Flow cytometry plots depicting pDC, cDC1, cDC2 or moDC co-cultured with activated- or naive CD4$^+$ T-cells. (**g**) Flow cytometry histograms depicting indicated markers expressed by DC under indicated conditions.

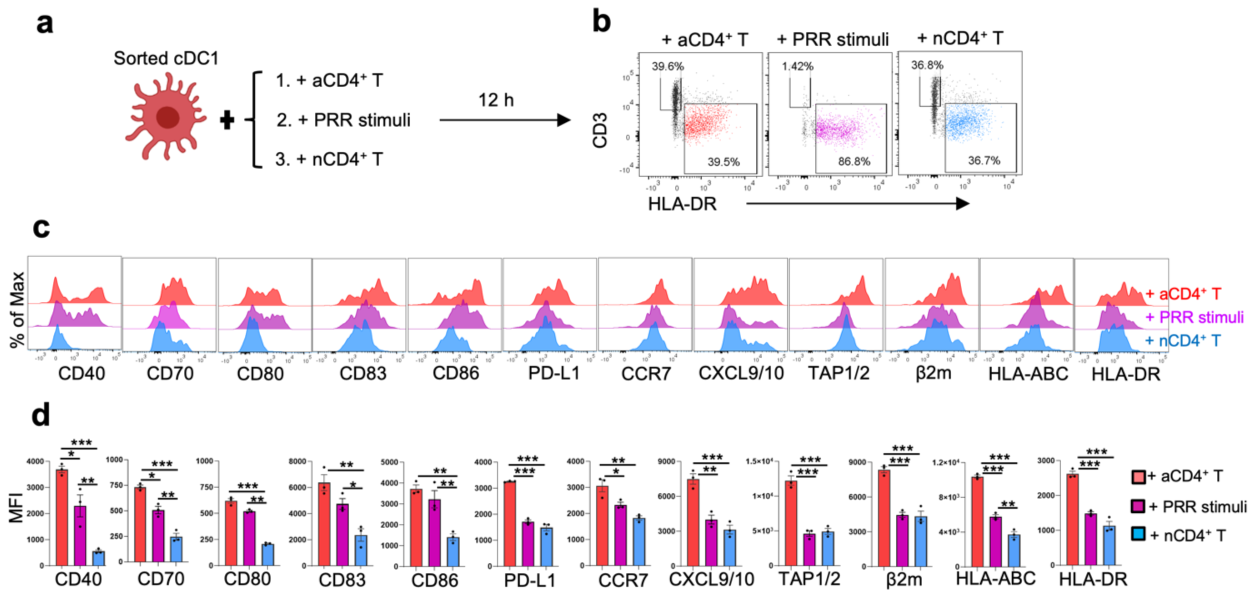

Supplementary Figure 6. Comparison of impact of CD4$^+$ T-cell help and PRR signals on cDC1. cDC1 were co-cultured with activated (a) or naïve (n)CD4$^+$ T-cells or a mixture of TLR3, -4, and -7/8 agonists, as specified in the Methods section. (**a**) Illustration of the experimental design. (**b**) Gating strategy for flow cytometric analysis of cDC1 after co-culture. (**c**) Flow cytometry histograms and (**d**) MFI quantifications for indicated markers expressed by cDC1 under indicated conditions. Data are pooled from three independent experiments (n=3) and are shown as means ± SEM. p<0.05*, p<0.01**, p<0.001*** (two-sided one-way ANOVA). (**e**) Heatmap depicting the expression of transcripts defined as the gene expression signature of human *in vitro* progenitor-derived XCR1$^+$ DC responding to PRR stimuli from Balan et al.[22] in each of our DC subsets co-cultured with activated (a) or naïve (n) CD4$^+$ T cells.

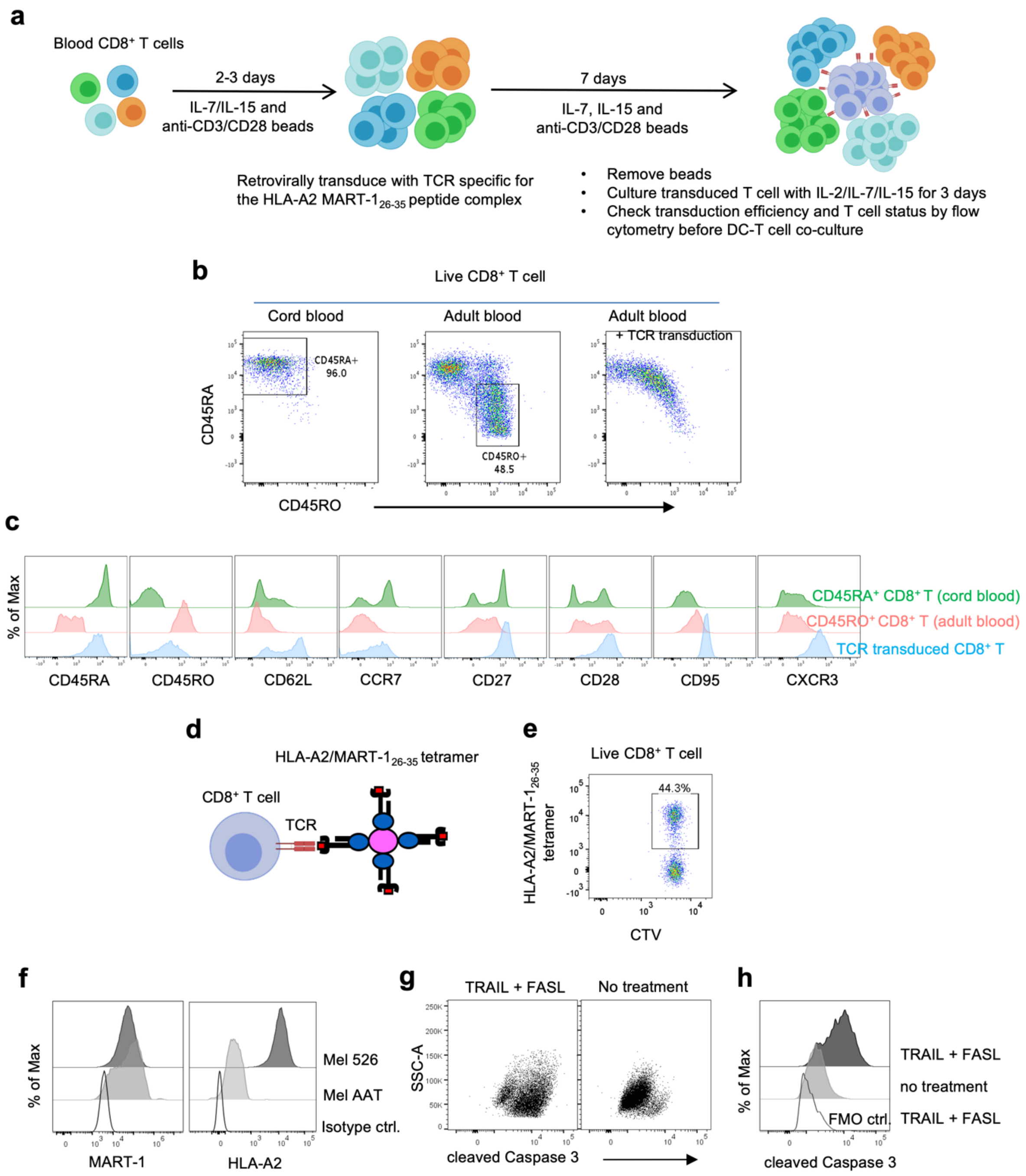

Supplementary Figure 7. Use of MART-1$_{26-35}$/HLA-A2 TCR transduced CD8$^+$ T-cells with T$_{SCM}$ phenotype and tumor cell-associated antigens for monitoring anti-tumor CTL response. (**a**) Schematic depiction of generation of MART-1-specific CD8$^+$ T-cells from PBMC of healthy donors. (**b**) Gating strategies for CD45RA$^+$ and CD45RO$^+$ CD8$^+$ T-cells from cord blood and adult blood respectively. (**c**) Expression of indicated markers identifying T$_{SCM}$ phenotype[26] of TCR transduced CD8$^+$ T-cells at day 2-3 after resting. CD45RA$^+$ CD8$^+$ naive T-cells (cord blood) and CD45RO$^+$ CD8$^+$ activated T-cells (adult blood) were used as controls. (**d**) Schematic depiction of tumor antigen-specific CD8$^+$ T-cell detection with HLA-A2/MART-1$_{26-35}$ tetramer. (**e**) Flow cytometry plot depicting CTV and tetramer staining. (**f**) Histograms depict in MFI the intracellular expression of MART-1 (left) and surface expression of HLA-A2 (right) by Mel 526 and Mel AAT cells. (**g, h**) Flow cytometry plots (**g**) and histogram (**h**) depict in MFI the intracellular expression of cleaved Caspase 3 by TRAIL/FASL treated Mel 526 cells, indicating apoptotic cell death.

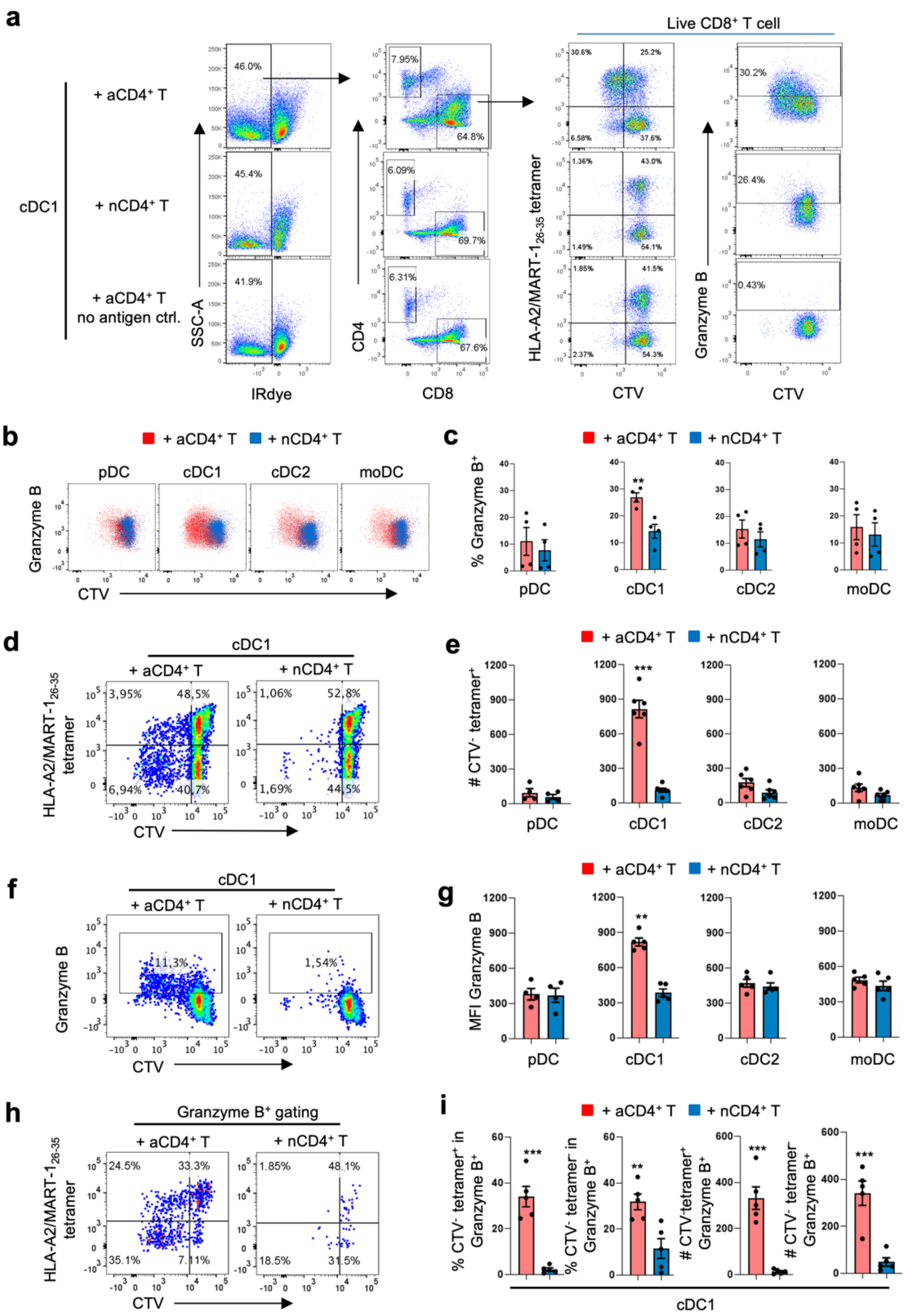

Supplementary Figure 8. Methodology for detecting CTL responses against tumor antigens in the *in vitro* priming system. pDC, cDC1, cDC2 and moDC were co-cultured with activated- (a) or naïve (n) $CD4^+$ T-cells and compared in their ability to cross-prime $CD8^+$ T-cells after loading with MART-1_{15-40} long peptide or Mel 526 cell debris. (**a**) Gating strategies for detecting a $CD8^+$ T-cell response in our *in vitro* priming system based on CTV dilution (proliferation) and intracellular Granzyme B staining (CTL differentiation). (**b, c**) Response to MART-1_{15-40} long peptide. (**b**) Flow cytometry plots depicting proliferation and Granzyme B expression by responding $CD8^+$ T cells. (**c**) Percentage (%) of Granzyme B^+ cells among $CD8^+$ T-cells. (**d-g**) Response to Mel 526 cell debris. Flow cytometry plots depicting (**d**) proliferation of $CD8^+$ T-cells based on CTV dilution and (**f**) CTL differentiation based on Granzyme B staining. (**e**) Number (#) of live MART-1-specific CTV^-CD8^+ T-cells. (**g**) Granzyme B expression (MFI) by $CD8^+$ T-cells. (**h**) Flow cytometry plots depicting proliferation of Granzyme B^+ $CD8^+$ T-cells based on CTV dilution. (**i**) Quantification of percentage (%) and number (#) of MART-1-specific ($tetramer^+$) CTV^- and non-MART-1-specific ($tetramer^-$) CTV^- $CD8^+$ T-cells within Granzyme B^+ cell gating. Data are pooled from four independent experiments (n=4) in (**c** and **i**), six independent experiments (n=6) in (**e**) and five independent experiments (n=5) in (**g**) and are shown as means ± SEM. Each experiment was performed with technical duplicates. $p<0.05$*, $p<0.01$**, $p<0.001$*** (two-sided Mann Whitney test).

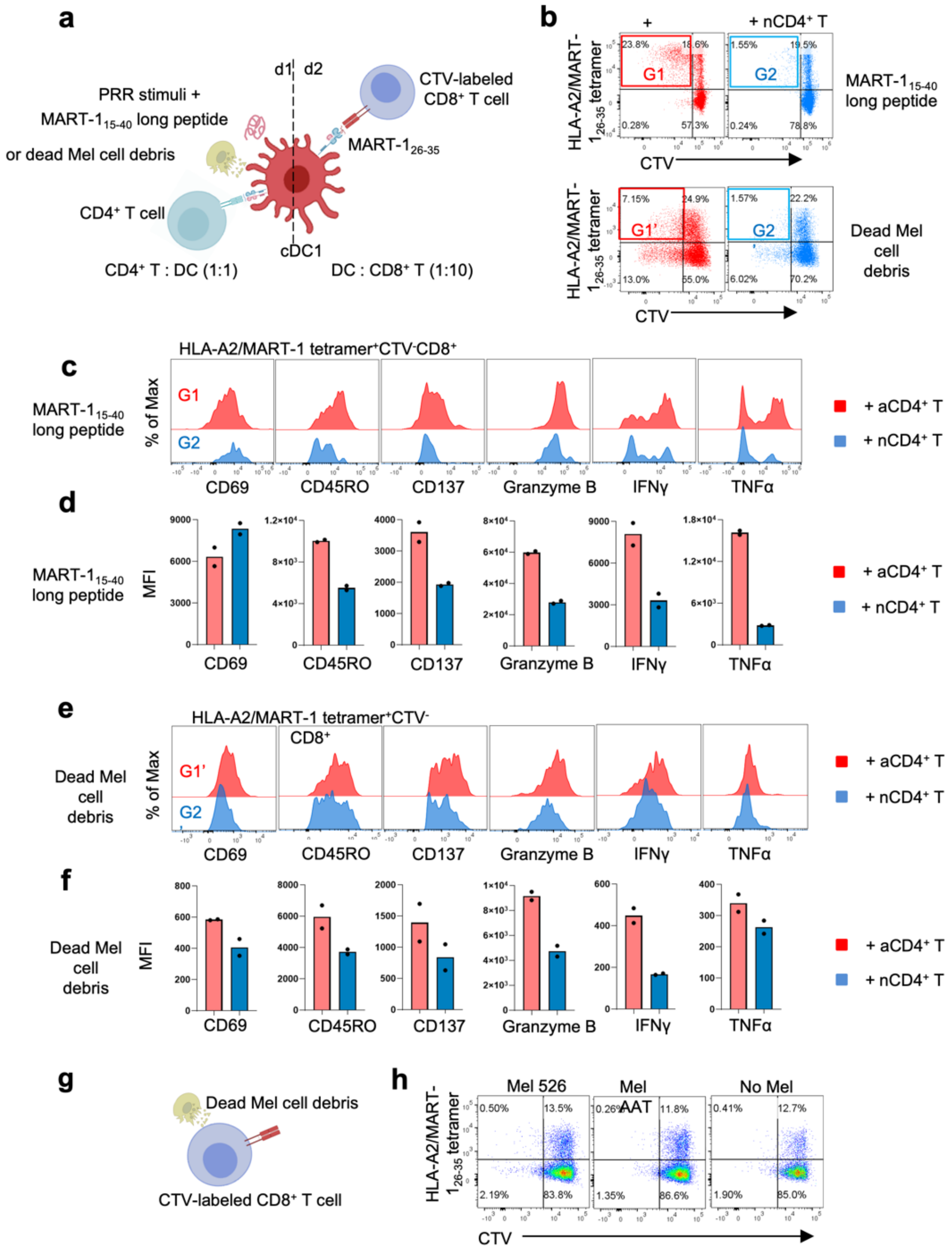

Supplementary Figure 9. Impact of CD4^{+} T-cell help on effector differentiation of CD8^{+} T-cells. (a) Sorted cDC1 were incubated with activated (a)- or naïve (n)- CD4^{+} T-cells and entered into the priming system with MART-1$_{15\text{-}40}$ long peptide or dead Mel AAT cell debris as antigen source. (**b**) Flow cytometry plots depicting the MART-1-specific CD8^{+} T-cell proliferation based on CTV dilution in response to MART-1 long peptide (upper panel) or dead Mel cell debris (lower panel). (**c**) Flow cytometry histograms and (**d**) MFI quantifications for indicated markers expressed by MART-1-specific (tetramer^{+}) proliferating (CTV^{-}) CD8^{+} T-cells in response to MART-1$_{15\text{-}40}$ long peptide under indicated conditions. (**e**) Flow cytometry histograms and (**f**) MFI quantifications for indicated markers expressed by MART-1-specific (tetramer^{+}) proliferating (CTV^{-}) CD8^{+} T-cells in response to dead Mel cell debris under indicated conditions. (**h**) Flow cytometry plots depicting the MART-1-specific CD8^{+} T-cell proliferation based on CTV dilution. Data are pooled from two independent experiments (n=2) in (**d, f**). (**g-h**) CTV-labeled CD8^{+} T-cells expressing the MART-1$_{26\text{-}35}$/HLA-A2-specific TCR were directly co-cultured with dead Mel cell debris without the presence of DC or CD4^{+} T-cells. (**g**) Schematic depiction of the co-culture. (**h**) Primary flow cytometry data representative of two independent experiments (n=2).

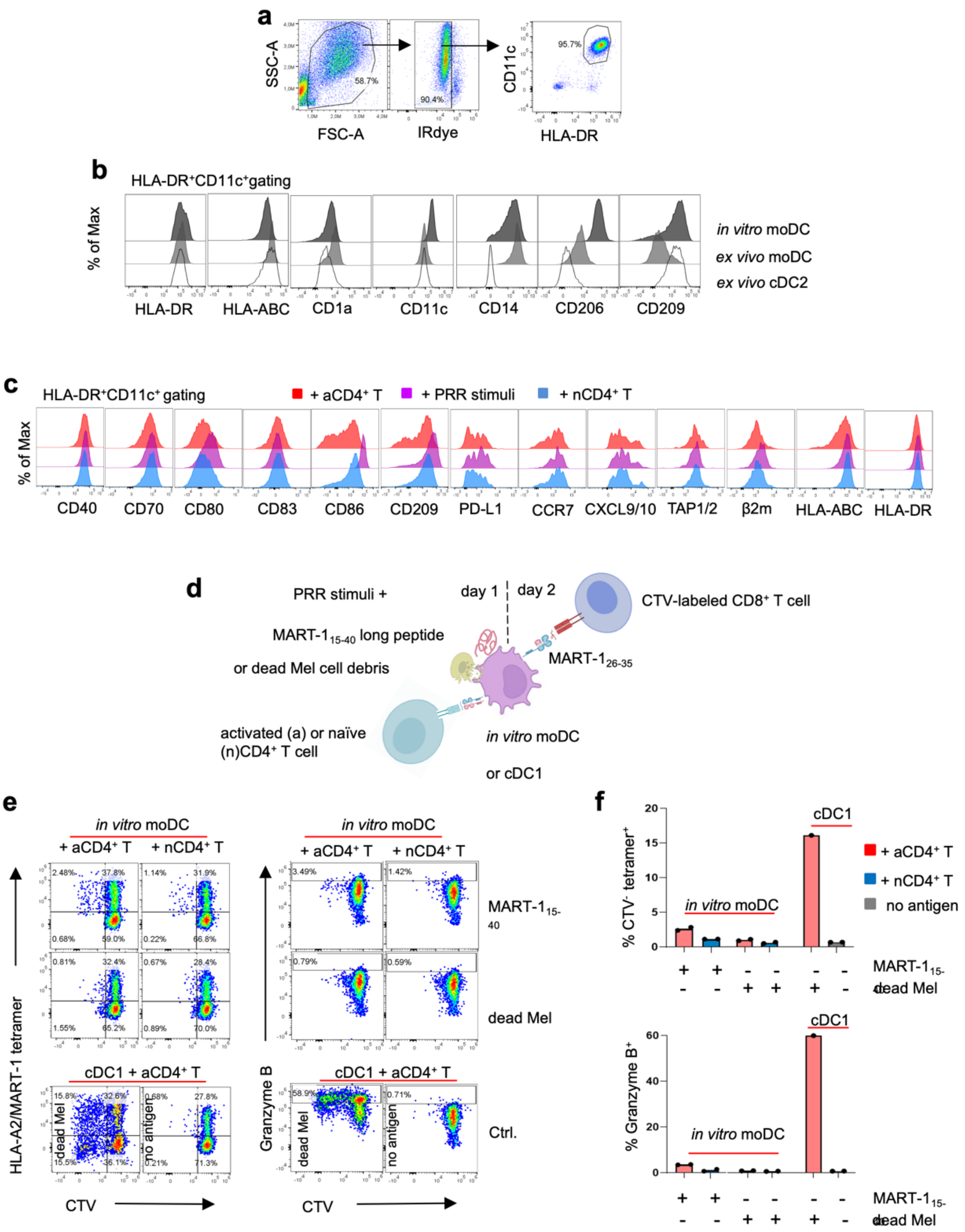

Supplementary Figure. 10. *In vitro* generated moDC do not evidently respond to CD4$^+$ T cell help. Monocytes were purified on CD14$^+$ phenotype by MACS magnetic sorting from fresh buffy coat of healthy donors and cultured for 7 days with GM-CSF (100 ng/ml) and IL-4 (50 ng/ml) to yield *in vitro* generated moDC. (**a**) Gating strategy. (**b**) Flow cytometry histograms depicting the expression of indicated markers on the indicated populations. (**c**) *In vitro* generated moDC were co-cultured with activated (a) or naïve (n)CD4$^+$ T-cells or combined PRR stimuli. Flow cytometry histograms depict the expression of indicated markers. FMO results indicated that the high CD70 signal was due to auto-fluorescence. Data are representative of two independent experiments (n=2), (**d**) cDC1 as a positive control and moDC as test population were incubated with activated (a)- or naïve (n)-CD4$^+$ T-cells and entered in the priming system with MART-1$_{15\text{-}40}$ long peptide or dead Mel AAT cell debris. (**e**) Flow cytometry plots depicting MART-1-specific CD8$^+$ T-cell proliferation based on CTV dilution (left panel) and CTL differentiation based on intracellular Granzyme B staining (right panel). (**f**) Quantification of the % MART-1$_{26\text{-}35}$/HLA-A2-specific (tetramer$^+$) cells within CTV-negative ($^-$) CD8$^+$ T-cells (upper panel) and the % Granzyme B$^+$ cells among CD8$^+$ T-cells (lower panel) under indicated conditions. Data are pooled from two independent experiments (n=2).

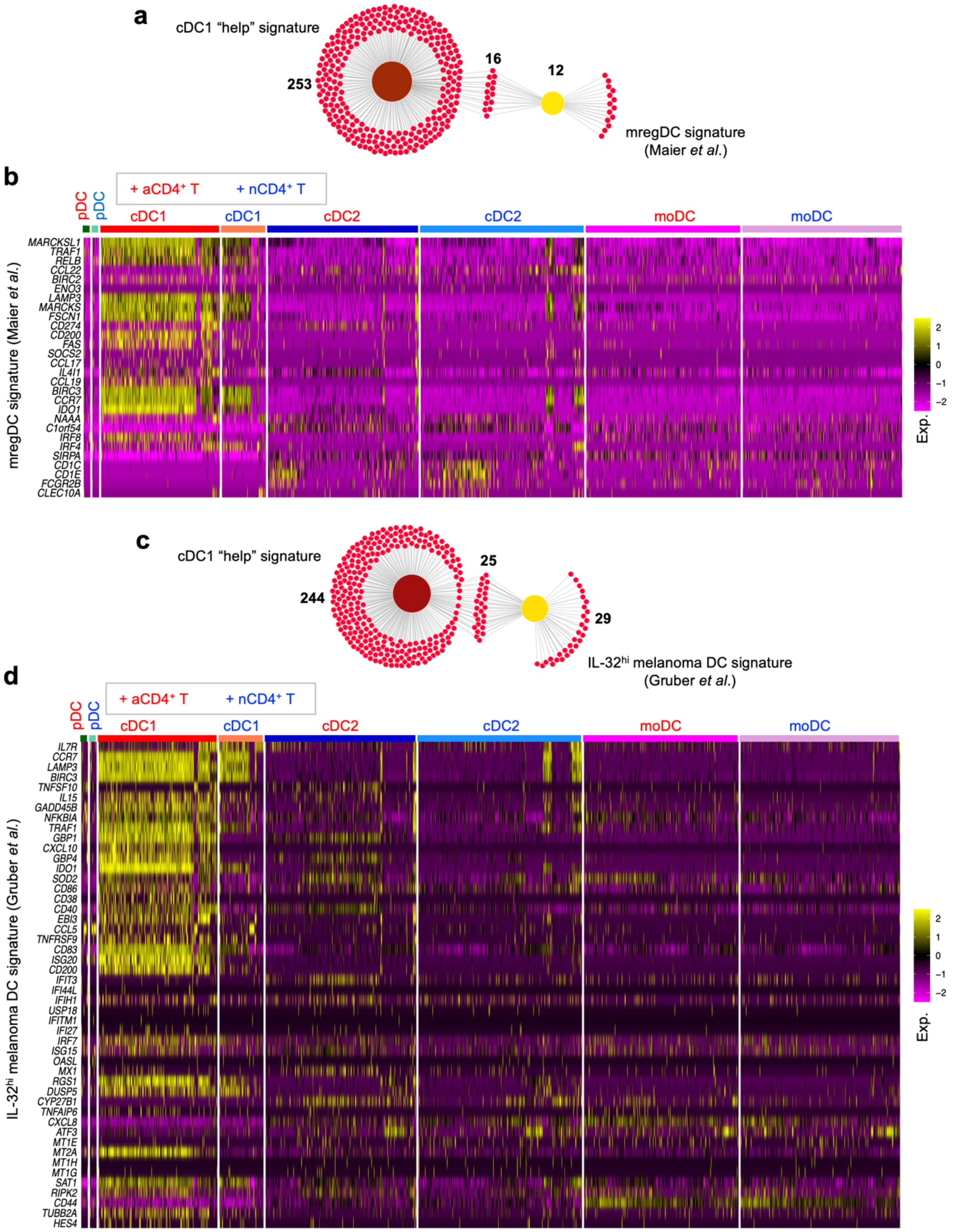

Supplementary Fig. 11. cDC1 help signature is related to tumor-infiltrating mregDC signature and mature DC signature in the IL-32^{hi} TME. Tumor-infiltrating mregDC[32] and mature DC signature in the IL-32^{hi} TME[33] were overlayed with cDC1 help signature. (**a**) Venn diagram depicting numbers of overlapping genes between the cDC1 help signature and tumor-infiltrating mregDC signature. (**b**) Heatmaps depicting expression of the tumor-infiltrating mregDC signature genes in each of our *ex vivo* DC subsets after co-culture with activated (a) or naïve (n) CD4^{+} T cells. (**c**) Venn diagram depicting numbers of overlapping genes between the cDC1 help signature and the IL-32^{hi} TME DC signature. (**d**) Heatmaps depicting expression of the IL-32^{hi} melanoma DC signature genes in each of our *ex vivo* DC subsets after co-culture with activated (a) or naïve (n) CD4^{+} T cells.

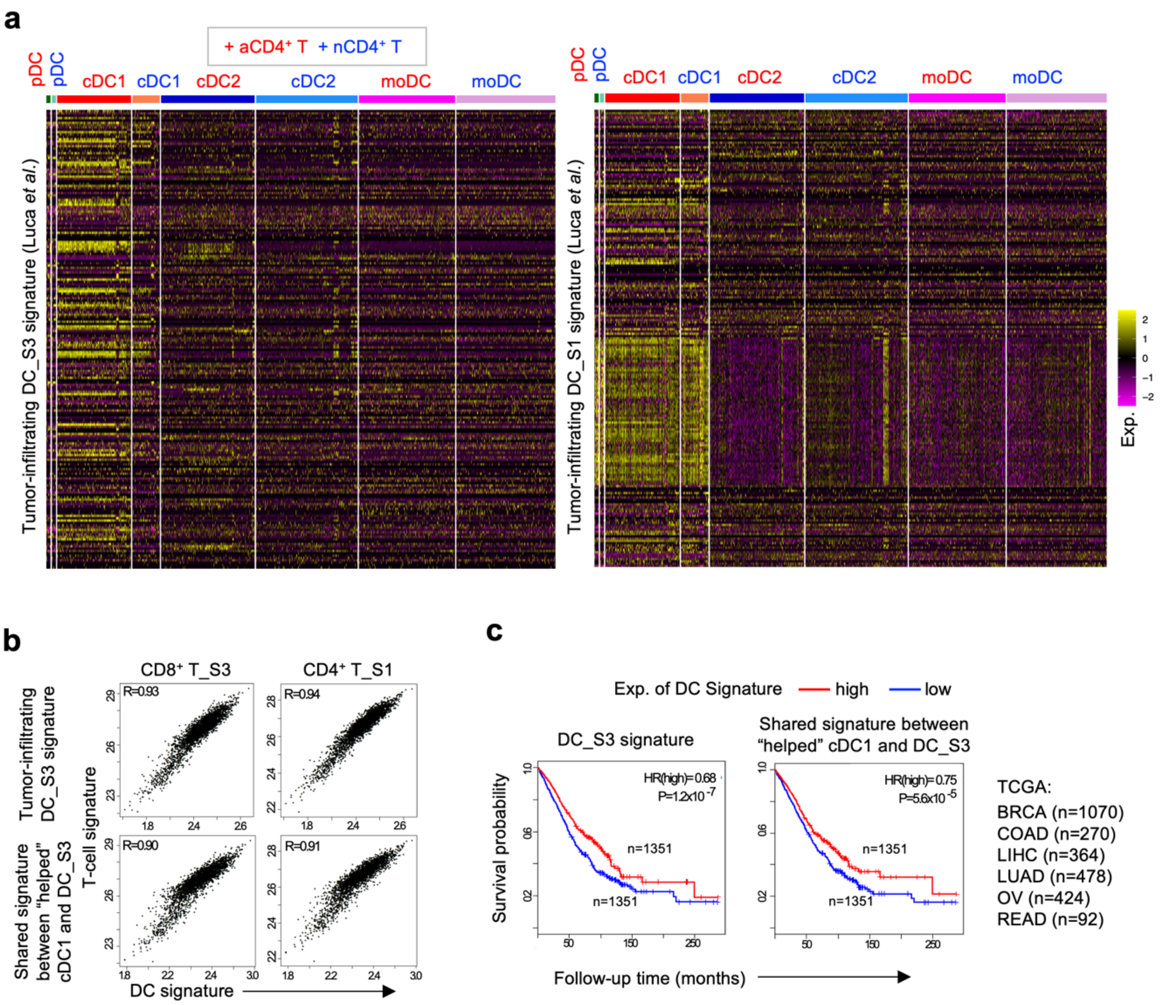

Supplementary Figure 12. Relationship between cDC1 help signature and tumor-infiltrating DC_S3 signature. Signature genes of 8 tumor-infiltrating DC states[18] were cross-compared with the cDC1 help signature. (**a**) Heatmaps depicting the expression of signature genes of tumor-infiltrating DC_S3 (222 genes) or DC_S1 (217 genes) in each of our *ex vivo* DC subsets co-cultured with activated (a) or naïve (n) $CD4^+$ T cells. (**b**) Pearson's correlations between tumor-infiltrating DC_S3 signature (upper panel), or shared signature between "helped" cDC1 and tumor-infiltrating DC_S3 (lower panel) and tumor infiltrating T-cell signatures from CE9 ($CD8^+$ T_S3, $CD4^+$ T_S1)). (**c**) Kaplan-Meier curves revealing overall prognostic/predictive impact of tumor-infiltrating DC_S3 signature (left), or shared signature between "helped" cDC1 and tumor-infiltrating DC_S3 (right) on patient's overall survival in TCGA BRCA, COAD, LIHC, LUAD, OV and READ cohorts (n=2702). High or low metagene expression subgroups of patients were based on a median expression cut-off. p-value was calculated using Log-rank test/Mantel-Cox test (statistical-significance threshold of $p < 0.05$).
