# Supplementary material for: CD4+ helper T cells endow cDC1 with cancer-impeding functions in the human tumor micro-environment
Source: Nat Commun. 2023 Jan 13;14:217. doi: 10.1038/s41467-022-35615-5 (PMC9839676; doi:10.1038/s41467-022-35615-5)
Supplement: Supplementary file 3 — Description of Additional Supplementary Files [file 41467_2022_35615_MOESM3_ESM.pdf]

Title: Supplementary Data 1.

Description: DC signatures from various studies. Data 1.1. cDC1 help signature genes used in Fig. 1, 4-5 and Supplementary Fig.12. **Data 1.2.** Top30 signature genes of DC & monocyte[20] (Villani et al. Science. 2017) used in Supplementary Fig.2. **Data 1.3.** Top30 signature genes of $CD14^{+}CD163^{+}$ DC[21] (Dutertre et al. Immunity. 2019) used in Supplementary Fig.2**. Data 1.4.** Signature of in vitro generated moDC[22] (Balan et al. JI. 2014) used in Supplementary Fig.2**. Data 1.5.** Mature DC signature in the IL-32hi TME33-34 (Jin et al. J.Transl.Med.2020; Grubar et al. JCI. 2020) used in Supplementary Fig.11. **Data 1.6.** mregDC signature[32] (Maier et al. Nature. 2020) used in Supplementary Fig.11**. Data 1.7.** Signature genes of tumor-infiltrating_DC3[17] (Gerhard et al. JEM. 2020) used in Fig.4-5**. Data 1.8.** Signature genes of tumor-infiltrating DC states[18] (Luca et al. Cell. 2021) used in Fig.4-5 and Supplementary Fig.12.

Title: Supplementary Data 2.

Description: T-cell signatures from various studies. Data 2.1. List of T-cell gene signatures[36] (Chaoentong & Trajanoski et al. Cell Reps. 2017) for TCGA datasets analysis used in Fig.5. **Data 2.2.** List of T-cell gene signatures[18] (Luca et al. Cell 2021) for TCGA datasets analysis used in Supplementary Fig.10

Title: Supplementary Data 3.
Description: List of all antibodies used in the current study and their dilutions.
