# Supplementary material for: CD4+ helper T cells endow cDC1 with cancer-impeding functions in the human tumor micro-environment
Source: Nat Commun. 2023 Jan 13;14:217. doi: 10.1038/s41467-022-35615-5 (PMC9839676; doi:10.1038/s41467-022-35615-5)
Supplement: Supplementary file 7 — Reporting Summary [file 41467_2022_35615_MOESM7_ESM.pdf]

nature portfolio

Corresponding author(s): Jannie Borst and Yanling Xiao

Last updated by author(s): Nov 25, 2022

Reporting Summary

Nature Portfolio wishes to improve the reproducibility of the work that we publish. This form provides structure for consistency and transparency in reporting. For further information on Nature Portfolio policies, see our Editorial Policies and the Editorial Policy Checklist.

Statistics

For all statistical analyses, confirm that the following items are present in the figure legend, table legend, main text, or Methods section.

n/a	Confirmed	
[ ]	[x]	The exact sample size (n) for each experimental group/condition, given as a discrete number and unit of measurement
[ ]	[x]	A statement on whether measurements were taken from distinct samples or whether the same sample was measured repeatedly
[ ]	[x]	The statistical test(s) used AND whether they are one- or two-sided<br>*Only common tests should be described solely by name; describe more complex techniques in the Methods section.*
[x]	[ ]	A description of all covariates tested
[x]	[ ]	A description of any assumptions or corrections, such as tests of normality and adjustment for multiple comparisons
[ ]	[x]	A full description of the statistical parameters including central tendency (e.g. means) or other basic estimates (e.g. regression coefficient) AND variation (e.g. standard deviation) or associated estimates of uncertainty (e.g. confidence intervals)
[ ]	[x]	For null hypothesis testing, the test statistic (e.g. F, t, r) with confidence intervals, effect sizes, degrees of freedom and P value noted<br>*Give P values as exact values whenever suitable.*
[x]	[ ]	For Bayesian analysis, information on the choice of priors and Markov chain Monte Carlo settings
[x]	[ ]	For hierarchical and complex designs, identification of the appropriate level for tests and full reporting of outcomes
[ ]	[x]	Estimates of effect sizes (e.g. Cohen's d, Pearson's r), indicating how they were calculated

Our web collection on statistics for biologists contains articles on many of the points above.

Software and code

Policy information about availability of computer code

Data collection	Flow cytometry data was collected using BD LSR Fortessa or BD FACSSymphony A5 SORP flow cytometer with BD FACSDIVA software.<br>Bulk mRNA sequencing data was collected on Illumina HighSeq 2500 sequencer.<br>scRNA sequencing data was collected on Illumina Nextseq 550 sequencer.
Data analysis	Flow cytometry data were analyzed using FlowJo software v10.7.2.<br>Bulk mRNA sequencing data were analyzed with TopHat software (v 2.1.0), Limma (v3.22.7), Qlucore Omics Explorer (v 3.7) and Ingenuity Pathway Analysis software (v. 52912811).<br>scRNA sequencing data were analyzed using Cell Ranger (v2.2.0), R (v 4.0) and Seurat package (v3.6.1).<br>GSEA was performed using GSEA software (v4.1.0) that can be downloaded via http://broadinstitute.org/gsea<br>Correlation and survival analysis using TCGA cohorts were performed using web based analysis tool (http://gepia2.cancer-pku.cn/#index).<br>Survival analysis using anti-PD1 clinical trial cohort was performed using Biomarker Evaluation' pipeline within a standardized TIDE computational workflow.<br>Statistical analysis were performed using Graphpad Prism (v.8).

For manuscripts utilizing custom algorithms or software that are central to the research but not yet described in published literature, software must be made available to editors and reviewers. We strongly encourage code deposition in a community repository (e.g. GitHub). See the Nature Portfolio guidelines for submitting code & software for further information.

Data

Policy information about availability of data

All manuscripts must include a data availability statement. This statement should provide the following information, where applicable:

- Accession codes, unique identifiers, or web links for publicly available datasets
- A description of any restrictions on data availability
- For clinical datasets or third party data, please ensure that the statement adheres to our policy

The bulk mRNA sequencing data utilized in Extended data Figure 1, 4 during the course of this study have been deposited in the GEO database with he accession number GSE218719. The cDC1 “help” signature has been listed in the extended data table1. The processed flow cytometry data are provided in the source data file. The scRNA sequencing data, other primary data and materials that support the findings of this study are available from the corresponding author upon request. Previously published tumor-infiltrating DC signatures can be accessed via DOI:10.1084/jem.20200264; 10.1172/jci.insight.138772 and 10.1016/j.cell.2021.09.014. Reactome database can be accessed via https://reactome.org/; TCGA cohorts used in survival analysis can be accessed via http://gepia2.cancer-pku.cn/#index.. Source data are provided with this paper.

Human research participants

Policy information about studies involving human research participants and Sex and Gender in Research.

Reporting on sex and gender	Gender information was not collected in the experiments of this study.
Population characteristics	All blood donors used in this study were healthy anonymous donors. We do not have information regarding the genotype, past or current diagnosis, treatments etc.
Recruitment	Blood donors were not actively recruited for this study.
Ethics oversight	The use of blood samples was approved by the internal ethical board of Sanquin, Amsterdam, The Netherlands.

Note that full information on the approval of the study protocol must also be provided in the manuscript.

Field-specific reporting

Please select the one below that is the best fit for your research. If you are not sure, read the appropriate sections before making your selection.

☒ Life sciences ☐ Behavioural & social sciences ☐ Ecological, evolutionary & environmental sciences

For a reference copy of the document with all sections, see nature.com/documents/nr-reporting-summary-flat.pdf

Life sciences study design

All studies must disclose on these points even when the disclosure is negative.

Sample size	No sample-size calculation was performed. Sample sizes were decided based on previous pilot experiments comparing cDC1 mediated T cell response with or without CD4 T-cell help. When using Mann-Whitney U test, we found when n= or >3, we observed significant difference between conditions.
Data exclusions	No data was excluded from the analysis
Replication	Each assays were performed three to six times as independent experiments with independent donors. within each experiment technical duplicates were used except where key molecules of cDC1 help signature were analyzed by flow cytometry. Not all attempts at replication were successful because cDC1 or pDC cell numbers were low for some donors or cells were unhealthy after sorting.
Randomization	No randomization was performed. In each experiment, all 4 ex vivo DC subsets were isolated from the same donor, and TCR transduced CD8 T-cells that are specific for MART-1/HLA-A2 were used to minimized the variation of between donors, as the transduction efficiencies were relatively consistent (between 30-50%).
Blinding	Investigators were not blinded to group allocation during data analysis, as experiments were performed and analyzed by the same people. However, data analysis was performed by two individuals independently and the results were consistent.

Reporting for specific materials, systems and methods

We require information from authors about some types of materials, experimental systems and methods used in many studies. Here, indicate whether each material, system or method listed is relevant to your study. If you are not sure if a list item applies to your research, read the appropriate section before selecting a response.

Materials & experimental systems

n/a	Involved in the study
[ ]	[x] Antibodies
[x]	[ ] Eukaryotic cell lines
[x]	[ ] Palaeontology and archaeology
[x]	[ ] Animals and other organisms
[x]	[ ] Clinical data
[x]	[ ] Dual use research of concern

Methods

n/a	Involved in the study
[x]	[ ] ChIP-seq
[ ]	[x] Flow cytometry
[x]	[ ] MRI-based neuroimaging

Antibodies

Antibodies used

Following antibodies were used:
From BioLegend:
CD1a-AF700 (clone HI149, cat#300120),
CD1c-PECY7 (clone L161, cat# 331515),
CD3-BV510 (clone OKT3, cat#317332),
CD4-BV785 (clone OKT4, cat# 317442),
CD8-AF700 (clone SK1, cat# 344724),
CD11c-PE (clone Bu15/3.9, cat# 337206),
CD14-BV785 (clone M5E2, cat#301840),
CD14-PB (clone 63D3, cat# 367122),
CD19-BV510 (clone HIB19, cat# 302242),
CD25-PE (clone BC96, cat# 302606),
CD28-PECY5 (clone CD28.2, cat# 302910),
CD40-BV711 (lone 5C3, cat#334334),
CD40L-PECY7 (clone 24-31, cat# 310832),
CD44-PE (clone BJ18, cat# 338808),
CD45RA-AF700 (clone HI100, cat# 304120),
CD62L-FITC (clone Dreg-56, cat# 304838),
CD69-BV510 (clone FN50, cat# 310936),
CD70-Percpcy5.5 (clone 113-16, cat# 355108),
CD80-BV785 (clone 2D10, cat# 305238),
CD83-BV421 (clone HB15e, cat# 305324),
CD86-BV510 (clone IT2.2, cat# 305432),
CD95-APCCY7 (clone Dx2, cat# 305636),
CD137-BV711 (clone 4B4-1, cat#309832),
CD141-APC (clone M80, cat# 344114),
CD206-PECY5 (clone 15-2, cat# 321108),
CD209-PECY7 (clone 9E9A8 ,cat#),
CD303-FITC/BV785 (clone 201A, cat# 354208/354222),
CCR7-Percpcy5.5 (clone G043H7, cat# 353220),
CXCL9-PE (clone J1015E10, cat# 357903),
CXCL10-PE (clone J034D6, cat# 519503),
CXCR3-PECY7 (clone G025H7, cat# 353720),
Granzyme B-PE (clone QA16A02, cat# 372208),
HLA-A2-PECY7 (clone BB7.2, cat# 343314),
HLA-ABC-FITC (clone W6/32, cat#311413),
HLA-DR-APCCY7 (clone L243, cat# 307618),
PD-L1-BV711 (clone 29E.2A3, cat# 329722),
β2-microglobulin-PE (clone A17082A, cat#395704),
IFNγ-PECY7 (clone B27, cat#506518),
TNFα-AF488(clone MAb11, cat#502915),
goat IgG Alexa Fluor 647 Isotype (cat # 403006),
purified mouse IgG1 k isotype ctrl antibody (cat# 401402).

From Abcam:
mouse anti-human Melan A (clone A103, cat#ab785)

From BD Biosciences:
CD27-BV650 (clone L128, cat# 563228),
CD45RO-PECF594 (clone UCHL1, cat# 562299),
HLA-DR-BV605 (clone G46-6, cat# 562845).

From Miltenyi Biotec:
CD141-APC (clone REA674, cat# 130-113-658).

From ImmunoTools:
CD8-APC (clone HIT8a, cat# 21810086).

From Cell Signaling Technology:
cleaved caspase 3-AF488 (clone D1751, cat# 9669S).

From Bioss:
rabbit anti-human TAP1 polyclonal antibody (cat# BS-2789R),
rabbit anti-human TAP2 polyclonal antibody (cat# BS-2374R).

From Thermo Fisher Scientific:
goat anti-rabbit IgG(H+L) AF488 secondary antibody (cat# A-11008),
goat anti-mouse IgG(H+L) AF467 secondary antibody (cat# A-21235).

APC-conjugated HLA-A2/MART-126-35 tetramers were provided by Peptide Facility in LUMC.
CD70 blocking antibody (CLB-2F2) was obtained from the laboratory of prof. R.A.W. van Lier (formerly at Sanquin, Amsterdam,The Netherlands).

From Sanquin:
CD3 (clone CLB-T3/4.E, cat# M1654)
CD28 (clone CLB-CD28/1, cat# M1650)

Validation	All antibodies have been validated by the manufacturers and in the lab of our colleagues where they are used extensively. The validation information for mouse anti-human Melan A can be found from https://www.abcam.com/melana-antibody-a103-ab785.html. The validation information for rabbit anti-human TAP1 can be found from https://www.biossusa.com/products/bs-2789r. The validation information for rabbit anti-human TAP1 can be found from https://www.biossusa.com/products/bs-2374r.The validation information for anti-CD3 and anti-CD28 antibodies can be found from https://pubmed.ncbi.nlm.nih.gov/32958937/.

Flow Cytometry

Plots

Confirm that:

- [ ] The axis labels state the marker and fluorochrome used (e.g. CD4-FITC).
- [x] The axis scales are clearly visible. Include numbers along axes only for bottom left plot of group (a 'group' is an analysis of identical markers).
- [ ] All plots are contour plots with outliers or pseudocolor plots.
- [x] A numerical value for number of cells or percentage (with statistics) is provided.

Methodology

Sample preparation	PBMC were isolated by standard Ficoll density gradient centrifugation, followed by cell sorting and co-culture as detailed in the materials and methods section.<br>For cell surface staining, cells were stained with flow cytometry staining buffer (BD Biosciences) on ice for 30 minutes. Following staining, cells were washed twice before harvesting in flow cytometry staining buffer.<br>For intracellular staining, protein transport inhibitor (BD GolgiPlug, BD Biosciences) was added for 3 hours before cells were stained. After cell surface staining, cells were fixed and permeabilized using the BD Cytofix/Cytoperm kit (BD Biosciences), according to the manufacturer's protocol. Cells were harvested in flow cytometry staining buffer.
Instrument	BD LSR Fortessa, BD FACSSymphony A5 SORP or Cytek Aurora spectral flow cytometer
Software	BD FACSDiva (v8), Flowjo (v10.7.2)
Cell population abundance	For cell sorting, 200x10^6 PBMC were used each time, CD19+ cells were depleted before sorting using CD19 magnetic MicroBeads (Miltenyi Biotech). From 200x10^6 PBMC, the general yields were 10,000-50,000 for cDC1, 50,000-200,000 for cDC2, 10-50,000 for pDC, 50,000-200,000 for MoDC, 2.5-10X10^6 for naive conventional CD4+ T cells, and 2-10x10^6 for CD8 T cells.

Gating strategy	The gating strategy for cell sorting is detailed in Extended Figure 1a and Extended Figure 5a-b.<br>The gating strategy for data analysis is detailed in Figure3c, Extended Figure 5e-f; Extended Figure 6b, Extended Figure 8a and Extended Figure10a.<br>For sorting, and analysis, cells were first gated: FSC-A/SSC-A, singlets SSC-A/SSC-H, and live cells using live/dead marker.<br>For sorting, after live/dead gating, T- and B-cells were excluded using CD3, CD19 and HLA-DR as detailed in the material and method section and in the supplementary information.<br>For DC response analysis, after live/dead gating, CD3 and HLA-DR were used to separate T-cells and DC.<br>For T-cell priming analysis, after live/dead gating, CD4 and CD8 were used to separate CD4- and CD8 T-cells, and tetramer was used to distinguish MART-1/HLA-A2 specific CD8 T cells, CTV was used to trace cell proliferation.

☒ Tick this box to confirm that a figure exemplifying the gating strategy is provided in the Supplementary Information.
