# Supplementary material for: CD4+ helper T cells endow cDC1 with cancer-impeding functions in the human tumor micro-environment
Source: Nat Commun. 2023 Jan 13;14:217. doi: 10.1038/s41467-022-35615-5 (PMC9839676; doi:10.1038/s41467-022-35615-5)
Supplement: Supplementary file 8 — Source files [file 41467_2022_35615_MOESM8_ESM.zip › Lei et al. Source files 20221208/Lei et al. Data files 20221208.docx]

**Gene set enrich analysis (GSEA)**

Gene Set Enrichment Analysis (GSEA) is a computational method that determines whether an a priori defined set of genes shows statistically significant, concordant differences between two biological states. The GSEA software is a joint project of UC San Diego and Broad Institute.

Step-by-step user guide:

1. The GSEA software can be downloaded using <http://broadinstitute.org/gsea>. Software can be installed by unzip the file.
2. Preparing the data: a gct. file with gene names and log2-fold change information and a cls. file with phenotype information are needed to run the GSEA. We have provided the gct. file with the 577 DEGs between cDC1 under “help” and “no-help” conditions, and the cls. file with phenotype information.
3. Open the software, load both the gct. file and cls. file. Click Run GSEA, then choose gene sets database, phenotype labels, chip platform accordingly. In the basic fields, max size and mi size for excluding larger and smaller sets can be decided, as well as the folder where results will be deposited. After all the parameters are filled, click run at the bottom of the panel.
4. Data visualization: after finishing running the analysis, results (GSEA plots like what’s demonstrated below) will be deposited in the folder of your choice.


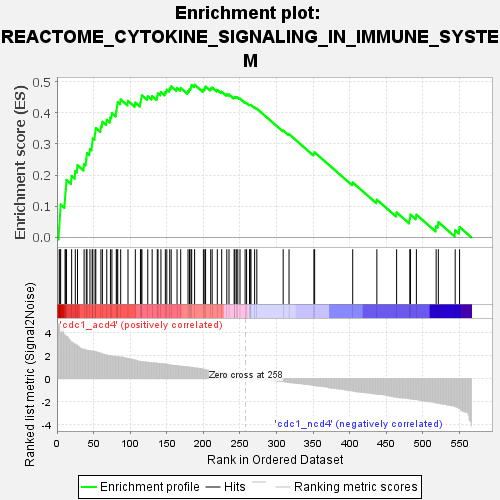


**Correlation, survival analysis using public datasets**

Correlation and survival analysis of TCGA cohorts were performed using GEPIA2 computational work-flow based on the UCSC Xena project (<http://xena.ucsc.edu>) that can be accessed using <http://gepia2.cancer-pku.cn/#index> . Survival analysis is based on log-rank hypothesis test (the Mantel–Cox statistical test) that also estimated the Cox-proportional hazard ratio (HR) and the 95% confidence intervals accompanied by a Kaplan-Meier (KM) plot. Survival analysis in immuno-oncology clinical trials was carried out using ‘Biomarker Evaluation’ pipeline within a standardized TIDE computational workflow, that can be accessed using <http://tide.dfci.harvard.edu/>. The predictive effects are calculated as z-score deduced using the Coxph statistical model. Data are represented as Kaplan–Meier curves.

Correlation analysis of TCGA cohorts:

1. In the Functions panel at the left side of the web page, choose ‘expression analysis’, then choose ‘correlation analysis’.
2. Upload the genes or signatures you want to use to perform correlation analysis, choose the statistical method for performing correlation analysis. Details in the signatures we used for performing correlation analysis are in supplementary table.
3. Choose the tissue type (e.g. tumor, normal tissue) from TCGA database you want to use to perform the correlation analysis.
4. Expected results are like plot demonstrated below.

Survival analysis of TCGA cohorts:

1. In the Functions panel at the left side of the web page, choose ‘expression analysis’, then choose ‘survival analysis’.
2. Upload the genes or signatures you want to use for the survival analysis. Details in the signatures we used for performing survival analysis are in supplementary table.
3. Choose the method, cutoff and cancer type you want to use to perform the survival analysis.
4. Expected results are like plot demonstrated below.

Survival analysis in immuno-oncology clinical trials

1. Access the ‘Biomarker Evaluation’ tab within the portal.
2. Upload the gene set, with comma and space separators, that you want to use for the survival analysis. Details in the signatures we used for performing survival analysis are in supplementary table.
3. Access the scatter plots for different biomarkers and your gene-set in the “survival” tab of the output for different cancer types.
4. Expected results are like plot demonstrated below for your specific gene set.
